# Analysis of genetic diversity by the SLAF-seq among the farmed *Onychostoma macrolepis* populations

**DOI:** 10.1186/s13104-024-06824-6

**Published:** 2024-06-20

**Authors:** Yuanhao Yang, Bang Han, Sien Wen, Fenggang Li, Hongbao Shen

**Affiliations:** 1 Fisheries research & Technology Extension Center of Shaanxi, Xi’an, China; 2 Yellow River Fisheries Research Institute, Chinese Academy of Fishery Sciences, Xi’an Shaanxi, China

**Keywords:** *Onychostoma macrolepis*, SLAF-seq, SNP, Genetic diversity

## Abstract

**Objective:**

The objective of this study was to examine the genetic diversity within and between farmed populations of *Onychostoma macrolepis*, and to establish a foundation for enhancing the genetic resources of breeding groups through the introduction of new individuals and crossbreeding. A total of 49 individuals were subjected to sequencing using Specific-Locus Amplified Fragment Sequencing (SLAF-seq), one of the restriction site-associated DNA sequencing technologies. The single nucleotide polymorphisms(SNPs)were identified to conduct the analyzation of phylogeny population structure, principal component and genetic diversity.

**Results:**

A total of 853,067 SNPs were identified. The results of the phylogenetic analysis revealed that each sample was genetically clustered into three distinct groups: ZhenPing (ZP), LanGao parents (LG), and their progeny population (LG-F1). Each population was observed to be clustered together. Analysis of population genetic diversity revealed that the observed heterozygosity (*Ho*) ranged from 0.200 to 0.230, the expected heterozygosity (*He*) ranged from 0.280 to 0.282, and the polymorphic information content (PIC) ranged from 0.228 to 0.230. These results indicate that the genetic diversity of the population is low and the signs of long-term interbreeding are obvious, but there are differences between the populations, and the genetic diversity of the population can be improved by hybridization in different regions.

## Introduction

*Onychostoma macrolepis* is a rare freshwater fish species in the Qinba Mountains, also known as money fish. It is a native Chinese omnivorous economic fish belonging to the Cyprinidae family, Barbinae subfamily, and Onychostoma genus [1]. It is mainly distributed in the middle and upper reaches of the Jialing River system and the Hanshui River system in China, coupled with its unique living environment and geographical distribution, known as a “living fossil” in water [2]. In recent years, due to overfishing, environmental pollution, and other reasons, the wild population of *O. macrolepis* has sharply decreased, and it has been included in the second level of China’s 2021 edition of the National Key Protected Wildlife List (limited to wild populations) [3]. In addition, due to the small scale of the breeding population of *O. macrolepis*, which is commonly bred within species for multiple generations, it may face the risk of germplasm degradation and genetic diversity, posing an enormous challenge to the healthy development of its breeding industry. Therefore, it is necessary to understand the genetic relationships and diversity information of different breeding populations, providing a reference for interspecies communication and variety optimization.

After years of development, genetic markers have entered an era of rapid development of molecular markers. The application of molecular marker methods can better explore the genetic background and molecular genetic laws of species, and provide technical support and theoretical basis for genetic breeding, strain identification and genetic diversity analysis of animals and plants [4]. Specific-locus amplified fragment sequencing (SLAF-seq) is a simplified genome sequencing technique that uses restriction endonucleases to cut DNA and sequence partial genomes. It has several distinguishing characteristics of deep sequencing to ensure accuracy of gene typing, effectively reduce sequencing costs, and pre-designed simplified representation scheme to optimize marker efficiency [5].

Genetic diversity is not only the material basis for species to adapt to environmental changes and continuous evolution, but also the genetic information basis for maintaining the fecundity of ethnic generations. The future of the ongoing breeding programs depends on the existing genetic diversity in the given population [6]. Thus, it was necessary to assess the extent of genetic diversity to maintain the health of the populations. SLAF-seq technology is widely used in the study of genetic diversity in marine habitat and plant populations [7, 8]. This study focuses on 49 samples of *O. macrolepis* from different populations. SLAF-seq technology was used to obtain a large number of polymorphic SLAF tags, and then single nucleotide polymorphism (SNP) sites were obtained on their tags, which can understand the genetic diversity of *O. macrolepis* breeding populations, to provide a theoretical basis for determining whether the breeding population needs to be introduced in a timely manner or interpopulation hybridization.

## Material and method

### Sample collection and genomic DNA extraction

In November 2022, a total of 49 *O. macrolepis* samples (Table 1) were collected from the breeding population in ZhenPing County, Shaanxi Province, as well as the parent population and their offspring population in LanGao County. The muscle tissue was extracted and frozen in liquid nitrogen. The genomic DNA of each sample was extracted using cetyltrimethyl annonium bromide (CTAB) protocol [9], and the DNA concentration was detected using Nanodrop. We confirm that all methods were performed in accordance with the Chinese Association for Laboratory Animal Sciences.

**Table 1:** Sampling information of *Onychostoma macrolepis*

Population	Sample Name	Sampling time	Sample number
ZhenPing (ZP)	ZP−1–ZP−17	2022−11−16	17
LanGao progeny (LG-F1)	LG−1–LG−17	2022−11−18	17
LanGao parents (LG)	LG−18–LG−32	2022−11−19	15

### Enzyme digestion library construction

Onychostoma_ Macropolis: GCA_ 012432095.1_ The ASM1243209v1 genome was used as the reference genome for electronic digestion prediction. The principle of selecting the optimal enzyme digestion scheme is to minimize the proportion of restriction fragments located in repeating sequence, as well as the degree of consistency between the length of restriction fragments and the specific experimental system [10]. The final decision was made to use HaeIII + HinCII enzyme digestion, and the genomic DNA of *O. macrolepis* was digested separately to obtain SLAF tags with a length of approximately 400 bp. Then, the ATP and dual-index sequencing adapter were added at the 3′and 5′end of the digested DNA products, respectively. PCR was performed and the products were purified using E.Z.N.A.H Cycle Pure Kit (Omega). The purified products were mixed and incubated with these two restricted enzymes again. After ligation of ATP, and Solexa adapter in the pair-end, the reaction products were purified using a Quick Spin column (Qiagen, Venlo, Netherlands), and segregated on a 2% agarose gel. These SLAFs were subjected to PCR to add barcode. The PCR products were re-purified and then prepared for paired-end sequencing on an Illumina HiSeq sequencing platform (Illumina, San Diego, CA, USA).

### Development of SNP tags

The raw reads were further processed with a bioinformatic pipelinetool, BMKCloud (www.biocloud.net) online platform. Raw data (raw reads) of fastq format were firstly processed through fastp software. In this step, clean data (clean reads) were obtained by removing reads containing adapter, reads containing ploy-N and low quality reads from raw data. At the same time, Q30, GC-content of the clean data were calculated. All the downstream analyses were based on clean data with high quality. The Dual index was used to identify the raw data obtained from sequencing and obtain the reads of each sample. Filter the sequencing reads connector and evaluate the quality to obtain clean reads. Clean reads were compared with the reference genome using BWA mem2 software [11]. The SNP/INDEL calling was performed using GATK (v3.8) [12] and SAMtools packages(v1.9.1) [13]. High consistency SNPs with a minor allele frequency (MAF) > 0.05 and integrity > 0.5 was retained.

### Phylogenetic analysis

Based on mutation detection of SNP-labeled data, MEGA X [14] software was used to construct phylogenetic trees for each sample using the Kimura 2-parameter model [15] with neighbor joining [16]. Bootstrap was repeated 1000 times. Using EIGENSOFT (v6.0) [17] software based on SNP data, principal component analysis was performed to obtain the clustering of the samples. Based on highly consistent SNPs, VCFtools (v0.1.15) [18] software was used to calculate various population genetic indicators, such as the polymorphism information content (PIC), average observed heterozygosity (*Ho*), and average expected heterozygosity (*He*).

## Result

### Sequencing data statistics

By performing restriction endonuclease prediction on the reference genome, a combination of HaeIII + HincII restriction endonucleases was selected to perform restriction endonuclease digestion on the genomic DNA of *O. macrolepis*. Sequences with a length of 314–414 bp were defined as SLAF tags. A total of 451.13 Mb reads were obtained in the experiment, with an average Q30 of 96.12% and an average GC content of 43.51%. Through bioinformatics analysis, a total of 274,172 SLAF tags were obtained from the genomes of *O. macrolepis* samples. The average sequencing depth of each SLAF tag in each sample was 15.48x, with 181,605 polymorphic SLAF tags. A total of 853,067 SNPs were developed for the *O. macrolepis* population.

### Evolutionary analysis of population systems

**Fig. 1A:**
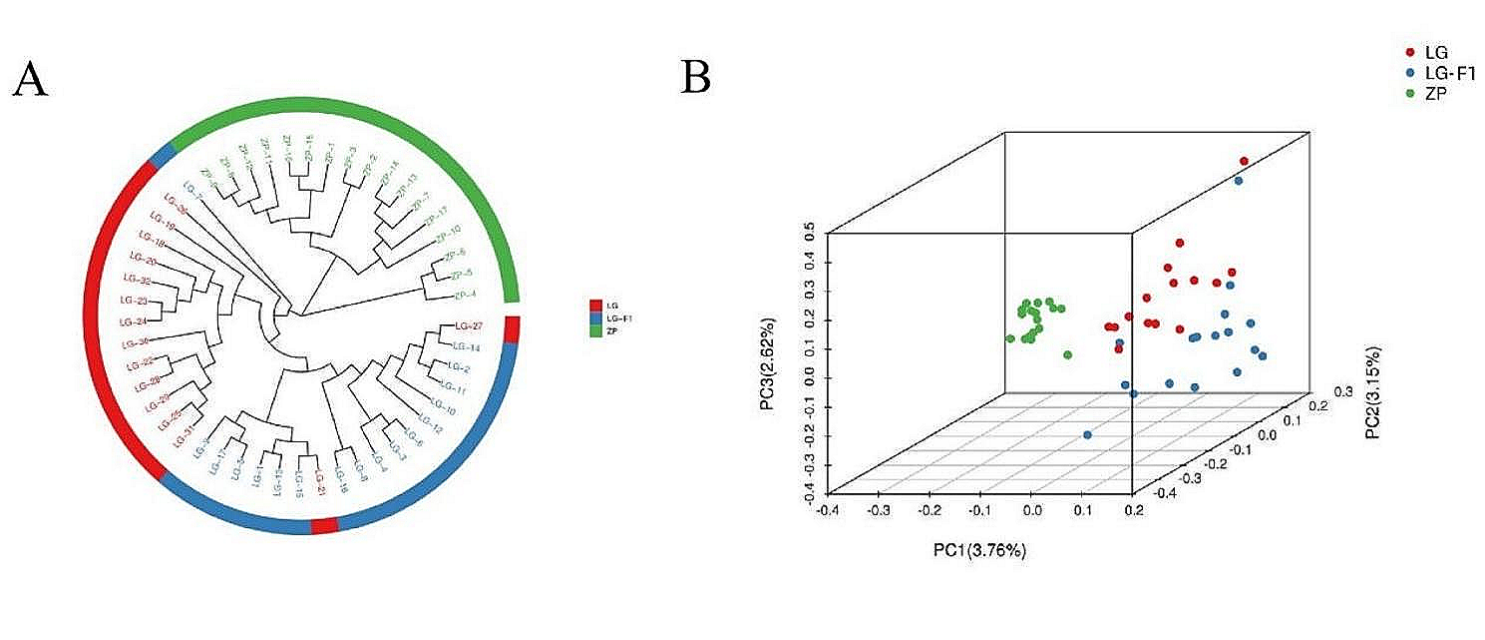
Phylogeny of the 49 *Onychostoma macrolepis* individuals; **1B**. Population PCA analysis. LG: LanGao parents population; LG-F1: LanGao progeny population; ZP: ZhenPing population

Based on mutation detection SNP data, highly consistent SNP loci (452,663) were obtained after filtering to construct phylogenetic trees for each sample (Fig. 1A). The phylogenetic evolutionary tree shows that each sample is genetically clustered into three clusters: ZP, LG, and LG-F1. Almost every population is individually clustered together, with the LG population crossing with its offspring, while the *O. macrolepis* population of ZP is individually clustered, indicating that its variety is relatively single and that there is almost no hybridization with the LG population.

To further understand the genetic relationships between populations, PCA was performed using highly consistent SNP loci (Fig. 1B). The three-dimensional clustering results showed that (the first, second, and third principal components were PC1, PC2, and PC3, respectively), the ZP population was clustered into a single cluster, and the LG and LG-F1 populations were relatively close.

### Analysis of population genetic diversity

By calculating parameters such as PIC, *Ho*, and *He* to evaluate the genetic diversity of each population, the results obtained based on SNP loci can be seen (Table 2), with *Ho* ranging from 0.200 to 0.230 and *He* ranging from 0.280 to 0.282. Among them, the *He* of the ZP populations is slightly higher than that of LG and LG-F1 populations, and its *Ho* is also slightly higher. The PIC of all three populations was between 0.228 and 0.230, with little difference between populations, indicating that the breeding population already had a low degree of polymorphism.

**Table 2:** Statistical values of genetic diversity among different populations of *Onychostoma macrolepis*

population	H_e_	H_o_	PIC
LG	0.280 ± 0.123	0.217 ± 0.201	0.228 ± 0.051
LG-F1	0.280 ± 0.116	0.200 ± 0.191	0.229 ± 0.059
ZP	0.282 ± 0.121	0.230 ± 0.184	0.230 ± 0.610

LG: LanGao parents population; LG-F1: LanGao progeny population; ZP: ZhenPing population; He: expected heterozygosity; Ho: observed heterozygosity; PIC: polymorphic information content; Data are represented as means ± SD

## Discussion

Genetic diversity analysis is a means to grasp the current situation of the germplasm resources of *O. macrolepis*. The genetic diversity information of *O. macrolepis* was evaluated from the SNP variation sites by bioinformatics technology, which has not been previously reported. Traditional molecular markers mainly include ISSR, RAPD, AFLP, etc. The disadvantages of traditional molecular markers include complex operability, low throughput, and low efficiency [19]. Liu [20] screened 15 ISSR primers for ISSR-PCR amplification of the genomic DNA of *Ophiocephalus argus* in three natural populations, and a total of 141 loci were detected, including 53 polymorphic loci, with a polymorphic rate of 37.59%. Du et al. [21] used RAPD technology to analyze the genetic diversity of *Bagarius yarrelli* population, and only 66 polymorphic loci were detected. By constructing the SLAF library, SLAF-seq can obtain high-quality SNP mutation site information, which is the most abundant, stable and efficient detection means [22]. Liu et al. [23] used SLAF-seq technology to study the populations of *Procambarus clarkii*, and obtained more than 350,000 SLAF tags and identified 741,147 SNPs. This experiment obtained a total of 274,172 SLAF tags, and developed 853,067 SNPs from the genomes of *O. macrolepis* samples. It can be seen that the results of this experiment demonstrate that SLAF-seq technology is superior to traditional molecular markers and is more suitable for marker development.

Regular testing of the genetic diversity of the breeding population can help reduce the risk of germplasm degradation. Compared with other economically cultivated varieties, the genetic diversity of the *O. macrolepis* is relatively low, mainly due to its inclusion in the national key protected wildlife list, coupled with the small number and scale of breeding populations, making it difficult to introduce [24, 25]. Regular testing of the genetic diversity of the *O. macrolepis* breeding population is an effective method to address the problem of germplasm degradation faced by its breeding industry [26, 27]. Liu et al. [23] used SLAF-seq to sequence the genomes of 14 populations of *Procambarus clarkii* in 5 provinces from china, including Hubei, Zhejiang, and Jiangsu. Genetic diversity indicators such as *Ho, He*, and PIC were obtained. The results showed that the *Ho* values ranged from 0.217 to 0.280, and *He* values ranged from 0.342 to 0.359, indicating the genetic diversity within the 14 populations was low and that the variety was single. Tian et al. [28] developed a large number of specific SNPs on polymorphic SLAF tags using SLAF-seq for genetic diversity analysis of different populations of Pinus bungeana. The results showed that the *Ho* values ranged from 0.353 to 0.424, and *He* values ranged from 0.358 to 0.391. In this study, the *Ho* and *He* values of the three populations were 0.200 to 0.230 and 0.280 to 0.282, respectively. From the *Ho* and *He* values of each population, the genetic diversity of the *O. macrocephalis* is relatively low, and it may face the risk of germplasm degradation. PIC reflects the genetic information capacity provided by SNP loci [29]. Ma [30]. conducted a study on the genetic diversity and phylogenetic relationship of two populations of *Schizothorax curvilabiatus* based on SLAF-seq technology. The PICs of two populations were 0.2877 and 0.2569, both of which were moderately polymorphic site. In this study, the PIC of the three populations of *O. macrolepis* were all less than 0.25, indicating that these populations were already had a low degree of polymorphism In addition, the observed heterozygosity of the three populations of *O. macrolepis* was also lower than the expected heterozygosity. It is speculated that the reason may be due to the long closed farming time, severe inbreeding, and insufficient communication with other breeding sites.

This study focuses on the *O. macrolepis* from three breeding populations. The cluster analysis results of the population showed that population from the ZP is a separate cluster, and the LG and LG-F1 population slightly intersect. Similar to phylogenetic trees, PCA yielded the same result: that is, the ZP population was clustered separately into one group. This can indicate that the phylogenetic relationships of the *O. macrolepis* are basically divided according to different geographical locations. In this study, it was found for the first time that there is a certain genetic difference between the LG population and the ZP population, which can be used as a selection for introduction in the future. At the same time, it was also found that the genetic diversity of *O. macrolepis* farmed populations in two different geographical locations was low, which may be related to long-term inbreeding and insufficient communication with populations in other regions. To ensure the genetic diversity of *O. macrolepis* in different regions, it is recommended to strengthen communication among populations in different regions, such as the LG and ZP populations with different geographical locations, which should be appropriately hybridized. Regular monitoring of population genetic diversity is necessary, which not only helps to protect the gene pool of *O. macrolepis* but also provides a timely and accurate grasp of the germplasm status, further promoting the sustainable and healthy development of the *O. macrolepis* farmed industry.

### Limitation

Our results were done with the limited numbers of samples and SNPs, and thus, the estimated statistics are expected to have variation. Terefore, these populations should also be tested using large sample sizes.

## Data Availability

The SLAF-seq reads have been deposited in the Sequence Read Archive (BioProject ID PRJNA1039559). The archived sequence data will be publicly available after publication.

## Abbreviations

SLAF-seq: Specific-locus amplified fragment sequencing
SNP: Single nucleotide polymorphisms
Ho: Observed heterozygosity
He: Expected heterozygosity
PIC: Polymorphic information content
